# Identification of potential modifier genes in Chinese patients with Wilson disease

**DOI:** 10.1093/mtomcs/mfac024

**Published:** 2022-03-31

**Authors:** Donghu Zhou, Siyu Jia, Liping Yi, Zhen Wu, Yi Song, Bei Zhang, Yanmeng Li, Xiaoxi Yang, Anjian Xu, Xiaojin Li, Wei Zhang, Weijia Duan, Zhenkun Li, Saiping Qi, Zhibin Chen, Qin Ouyang, Jidong Jia, Jian Huang, Xiaojuan Ou, Hong You

**Affiliations:** Experimental Center, Beijing Friendship Hospital, Capital Medical University, Beijing 100050, China; Clinical Research Center for Rare Liver Diseases, Capital Medical University, Beijing, China; National Clinical Research Center for Digestive Diseases, On behalf of China Registry of Genetic/Metabolic Liver Diseases (CR-GMLD) Group, Beijing, China; Experimental Center, Beijing Friendship Hospital, Capital Medical University, Beijing 100050, China; Clinical Research Center for Rare Liver Diseases, Capital Medical University, Beijing, China; National Clinical Research Center for Digestive Diseases, On behalf of China Registry of Genetic/Metabolic Liver Diseases (CR-GMLD) Group, Beijing, China; Liver Research Center, Beijing Friendship Hospital, Capital Medical University, Beijing Key Laboratory of Translational Medicine on Liver Cirrhosis, Beijing, China; Clinical Research Center for Rare Liver Diseases, Capital Medical University, Beijing, China; National Clinical Research Center for Digestive Diseases, On behalf of China Registry of Genetic/Metabolic Liver Diseases (CR-GMLD) Group, Beijing, China; Liver Research Center, Beijing Friendship Hospital, Capital Medical University, Beijing Key Laboratory of Translational Medicine on Liver Cirrhosis, Beijing, China; Clinical Research Center for Rare Liver Diseases, Capital Medical University, Beijing, China; National Clinical Research Center for Digestive Diseases, On behalf of China Registry of Genetic/Metabolic Liver Diseases (CR-GMLD) Group, Beijing, China; Experimental Center, Beijing Friendship Hospital, Capital Medical University, Beijing 100050, China; Clinical Research Center for Rare Liver Diseases, Capital Medical University, Beijing, China; National Clinical Research Center for Digestive Diseases, On behalf of China Registry of Genetic/Metabolic Liver Diseases (CR-GMLD) Group, Beijing, China; Experimental Center, Beijing Friendship Hospital, Capital Medical University, Beijing 100050, China; Clinical Research Center for Rare Liver Diseases, Capital Medical University, Beijing, China; National Clinical Research Center for Digestive Diseases, On behalf of China Registry of Genetic/Metabolic Liver Diseases (CR-GMLD) Group, Beijing, China; Experimental Center, Beijing Friendship Hospital, Capital Medical University, Beijing 100050, China; Clinical Research Center for Rare Liver Diseases, Capital Medical University, Beijing, China; National Clinical Research Center for Digestive Diseases, On behalf of China Registry of Genetic/Metabolic Liver Diseases (CR-GMLD) Group, Beijing, China; Experimental Center, Beijing Friendship Hospital, Capital Medical University, Beijing 100050, China; Clinical Research Center for Rare Liver Diseases, Capital Medical University, Beijing, China; National Clinical Research Center for Digestive Diseases, On behalf of China Registry of Genetic/Metabolic Liver Diseases (CR-GMLD) Group, Beijing, China; Experimental Center, Beijing Friendship Hospital, Capital Medical University, Beijing 100050, China; Clinical Research Center for Rare Liver Diseases, Capital Medical University, Beijing, China; National Clinical Research Center for Digestive Diseases, On behalf of China Registry of Genetic/Metabolic Liver Diseases (CR-GMLD) Group, Beijing, China; Experimental Center, Beijing Friendship Hospital, Capital Medical University, Beijing 100050, China; Clinical Research Center for Rare Liver Diseases, Capital Medical University, Beijing, China; National Clinical Research Center for Digestive Diseases, On behalf of China Registry of Genetic/Metabolic Liver Diseases (CR-GMLD) Group, Beijing, China; Liver Research Center, Beijing Friendship Hospital, Capital Medical University, Beijing Key Laboratory of Translational Medicine on Liver Cirrhosis, Beijing, China; Clinical Research Center for Rare Liver Diseases, Capital Medical University, Beijing, China; National Clinical Research Center for Digestive Diseases, On behalf of China Registry of Genetic/Metabolic Liver Diseases (CR-GMLD) Group, Beijing, China; Liver Research Center, Beijing Friendship Hospital, Capital Medical University, Beijing Key Laboratory of Translational Medicine on Liver Cirrhosis, Beijing, China; Clinical Research Center for Rare Liver Diseases, Capital Medical University, Beijing, China; National Clinical Research Center for Digestive Diseases, On behalf of China Registry of Genetic/Metabolic Liver Diseases (CR-GMLD) Group, Beijing, China; Experimental Center, Beijing Friendship Hospital, Capital Medical University, Beijing 100050, China; Clinical Research Center for Rare Liver Diseases, Capital Medical University, Beijing, China; National Clinical Research Center for Digestive Diseases, On behalf of China Registry of Genetic/Metabolic Liver Diseases (CR-GMLD) Group, Beijing, China; Experimental Center, Beijing Friendship Hospital, Capital Medical University, Beijing 100050, China; Clinical Research Center for Rare Liver Diseases, Capital Medical University, Beijing, China; National Clinical Research Center for Digestive Diseases, On behalf of China Registry of Genetic/Metabolic Liver Diseases (CR-GMLD) Group, Beijing, China; Experimental Center, Beijing Friendship Hospital, Capital Medical University, Beijing 100050, China; Clinical Research Center for Rare Liver Diseases, Capital Medical University, Beijing, China; National Clinical Research Center for Digestive Diseases, On behalf of China Registry of Genetic/Metabolic Liver Diseases (CR-GMLD) Group, Beijing, China; Experimental Center, Beijing Friendship Hospital, Capital Medical University, Beijing 100050, China; Clinical Research Center for Rare Liver Diseases, Capital Medical University, Beijing, China; National Clinical Research Center for Digestive Diseases, On behalf of China Registry of Genetic/Metabolic Liver Diseases (CR-GMLD) Group, Beijing, China; Liver Research Center, Beijing Friendship Hospital, Capital Medical University, Beijing Key Laboratory of Translational Medicine on Liver Cirrhosis, Beijing, China; Clinical Research Center for Rare Liver Diseases, Capital Medical University, Beijing, China; National Clinical Research Center for Digestive Diseases, On behalf of China Registry of Genetic/Metabolic Liver Diseases (CR-GMLD) Group, Beijing, China; Experimental Center, Beijing Friendship Hospital, Capital Medical University, Beijing 100050, China; Liver Research Center, Beijing Friendship Hospital, Capital Medical University, Beijing Key Laboratory of Translational Medicine on Liver Cirrhosis, Beijing, China; Clinical Research Center for Rare Liver Diseases, Capital Medical University, Beijing, China; National Clinical Research Center for Digestive Diseases, On behalf of China Registry of Genetic/Metabolic Liver Diseases (CR-GMLD) Group, Beijing, China; Liver Research Center, Beijing Friendship Hospital, Capital Medical University, Beijing Key Laboratory of Translational Medicine on Liver Cirrhosis, Beijing, China; Clinical Research Center for Rare Liver Diseases, Capital Medical University, Beijing, China; National Clinical Research Center for Digestive Diseases, On behalf of China Registry of Genetic/Metabolic Liver Diseases (CR-GMLD) Group, Beijing, China; Experimental Center, Beijing Friendship Hospital, Capital Medical University, Beijing 100050, China; Liver Research Center, Beijing Friendship Hospital, Capital Medical University, Beijing Key Laboratory of Translational Medicine on Liver Cirrhosis, Beijing, China; Clinical Research Center for Rare Liver Diseases, Capital Medical University, Beijing, China; National Clinical Research Center for Digestive Diseases, On behalf of China Registry of Genetic/Metabolic Liver Diseases (CR-GMLD) Group, Beijing, China

**Keywords:** Wilson disease, ATP7B, mutation, modifier gene, CTR2, copper uptake

## Abstract

The mutations in modifier genes may contribute to some inherited diseases including Wilson disease (WD). This study was designed to identify potential modifier genes that contribute to WD. A total of 10 WD patients with single or no heterozygous *ATP7B* mutations were recruited for whole-exome sequencing (WES). Five hundred and thirteen candidate genes, of which the genetic variants present in at least two patients, were identified. In order to clarify which proteins might be involved in copper transfer or metabolism processes, the isobaric tags for relative and absolute quantitation (iTRAQ) was performed to identify the differentially expressed proteins between normal and CuSO_4_-treated cell lines. Thirteen genes/proteins were identified by both WES and iTRAQ, indicating that disease-causing variants of these genes may actually contribute to the aberrant copper ion accumulation. Additionally, the c.86C > T (p.S29L) mutation in the *SLC31A2* gene (coding CTR2) has a relative higher frequency in our cohort of WD patients (6/191) than reported (0.0024 in gnomAD database) in our healthy donors (0/109), and CTR2^S29L^ leads to increased intracellular Cu concentration and Cu-induced apoptosis in cultured cell lines. In conclusion, the WES and iTRAQ approaches successfully identified several disease-causing variants in potential modifier genes that may be involved in the WD phenotype.

## Graphical Abstract

**Graphical Abstract:**
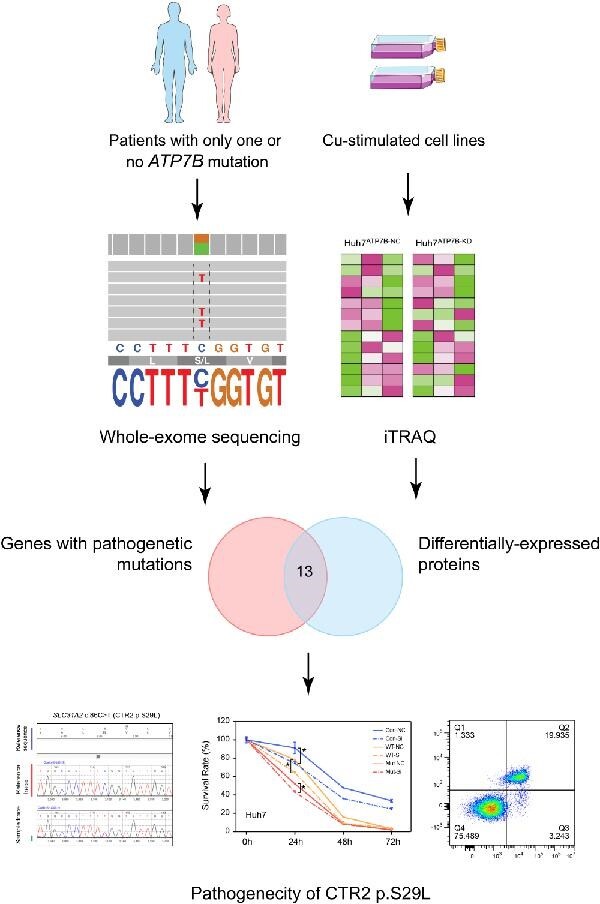
Identification of modifier genes of Wilson disease.

## Introduction

Wilson disease (WD, OMIM entry: 277 900) is a rare autosomal recessive genetic disorder associated with various mutations in the *ATP7B* gene. It affects 1 in 30 000 individuals with a carrier frequency of 1 in every 90, the common symptoms including abdominal pain, jaundice, personality change, etc. The diagnostic algorithm is based on a diagnostic index (“Leipzig” score) proposed by an expert panel^1^. This score includes clinical findings (Kayser–Fleischer corneal ring), biochemical findings (low serum copper and ceruloplasmin concentrations, and increased urinary copper excretion), and molecular features (detection of biallelic *ATP7B* pathogenic variants). The mainstay therapy for WD is copper chelation therapy with penicillamine or trientine.

Previous research has shown the genotype of patients with WD varies between different populations. According to our previous research, in China, about 66.2% of patients with WD have compound heterozygous variants, and only 12.3% are homozygotes.^2^ However, recent studies have indicated that approximately 12–20% of patients with WD have only one or no heterozygous mutation, whether from China or from Western countries. These patients also have some typical WD phenotypes such as increased urinary copper excretion and low serum ceruloplasmin concentration.^2,3^

To date, molecular testing methods used to diagnose WD in the clinic have mainly relied on Sanger sequencing.^4,5^ However, this approach is tedious and ineffective when sequencing large DNA fragments or searching for disease-causing variants in potential modifier genes. Whole-exome sequencing (WES) provides an efficient approach for identifying large fragment deletions and rare mutations^6,7^, and provides an accurate way to differentiate between systemic errors and actual biological variations.^8,9^ The application of WES has already exhibited superiority to Sanger sequencing in the clinical genetic diagnosis of several kinds of inherited diseases.^10–12^

A modifier gene is a gene that, when mutated, does not produce an effect on its own, but may produce or enhance clinical manifestations when coupled with another genetic mutation.^13^ Several genes have been proposed as potential modifier genes for WD, including *ATOX1* (antioxidant copper chaperone 1)^14^, *COMMD1* (copper metabolism domain-containing 1)^15^, *XIAP* (X-linked inhibitor of apoptosis)^16^, and *HFE* (hemochromatosis gene).^17^ However, none of these candidates has been confirmed by additional research.

Copper is an essential micronutrient required for oxygen-dependent enzymes. However, copper in excess is a noxious agent due to the oxidative capacity in biological systems. The opposing forces is balanced by chaperones and membrane transporters, which control copper uptake and moving copper across membranes.^18,19^ The high-affinity Cu transporter, copper transport protein, CTR1, is responsible for the majority (∼70%) of Cu intake.^20^ Upon entry, Cu may bind transiently to glutathione and be retrieved by Cu chaperones. Cytosolic Cu chaperone for SOD1 facilitates Cu incorporation, as well as the formation of a disulfide bond.^21^ ATOX1 binds Cu(I) within the Cys–Gly–Gly–Cys metal-binding site, increasing the delivery of copper to the secretory pathway.^22^ The P-type ATPase transporters, ATP7A and ATP7B, regulate cytoplasmic copper by pumping them out of cells or into the endomembrane system. In the *trans*-Golgi network (TGN), ATP7B transfers Cu to the ceruloplasmin, which is then secreted across the basolateral membrane. Cu elevation triggers trafficking of ATP7B from the TGN toward the apical membrane, thus facilitating export of excess Cu.^19^

The CTR1 and CTR2 copper transporters are influx Cu(I) transporters in mammalian cells. CTR1 has a much higher affinity for copper than CTR2 and largely resides in the plasma membrane. CTR2 has a lower affinity for copper and is mostly associated with vesicular structures inside the cell. Only part of the CTR2 resides in the plasma membrane^23^, but it plays a role in stabilizing a truncated form of Ctr1 lacking the extracellular domain. Retention of this domain in mice or cells lacking Ctr2 enhances copper uptake.^24^ Generally, CTR1 mobilizes Cu(I) from the vacuole when Cu(I) is scarce, but the function of CTR2 is far from fully understood. For the WD patients with typical clinical symptoms but only single or no heterozygous *ATP7B* mutation, it is plausible to hypothesize that the copper transporters (CTR1 and/or CTR2) might act as modifier genes to influence the WD phenotype.

## Patients and methods

### Diagnostic criteria

A total of 201 patients with WD were recruited from the China Registry of Genetic/Metabolic Liver Diseases (CR-GMLD) Group. After laboratory and genetic tests, diagnosis of WD was established in accordance with the Leipzig score (≥4), and 10 patients with only one or no *ATP7B* mutation were selected for WES. An additional cohort of 191 patients with WD was recruited to determine the frequency of the CTR2 p.S29L mutation.

### Sanger sequencing

Candidate pathogenic mutations in patients with WD and their immediate family members were identified by Sanger sequencing using an ABI 3730 analyser (Applied Biosystem). All of the 21 exons and at least 50 bp of intronic flanking sequences of the *ATP7B* gene were amplified, using primers reported previously.^2^ Sites of variation were identified through comparing DNA sequences with the corresponding GenBank reference sequences (GRCh38, https://www.ncbi.nlm.nih.gov/genbank/) using Mutation Surveyor software.

### Whole-exome sequencing

#### DNA library preparation and sequencing

Peripheral blood (approximately 2 mL) was collected from the patients, and genomic DNA was extracted using the TIANamp Blood DNA Kit (Tiangen Biotech Co., China). DNA libraries were prepared according to Illumina's (USA) protocol. In brief, 3 μg of genomic DNA was fragmented by sonication (Covaris S2, USA). An “A” was then ligated to the 3′-end of the fragmented DNA. The sample was size-selected aiming to obtain segments of 150–∼250 bp for PCR amplification. Enriched libraries were then sequenced using an Illumina NextSeq 500 sequencer (Illumina) for paired-end reads of 150 bp.

#### Data analysis

Low-quality variations were filtered out using a quality score ≥20 and the Burrows-Wheeler Alignment tool software package^25^ was used to align clean reads to the reference human genome (GRCh37). Single-nucleotide polymorphisms (SNPs) were identified using the SOAPsnp program (http://soap.genomics.org.cn/soapsnp.html), and insertions or deletions (InDels) were identified using GATK (http://www.broadinstitute.org/gsa/wiki/index.php/Home_Page). The identified SNPs and InDels were annotated using the Exome-assistant program (http://122.228.158.106/exomeassistant). SNPs and InDels with frequencies >0.01 in 1000 Genome, ESP6500, ExAC_ALL, and ExAC_EAS were removed. Nonsynonymous variants were evaluated by Ployphen-2 (http://genetics.bwh.harvard.edu/pph2/), SIFT (http://sift.bii.a-star.edu.sg/), and MutationTaster (http://www.mutationtaster.org/) programs to predict their pathogenicity.

#### Cell culture

HEK-293T, BEL-7402, and Huh7 cell lines were purchased from National Collection of Authenticated Cell Cultures (Shanghai, China), and incubated at 37°C with 5% CO_2_ in a humidified atmosphere. The cell lines were maintained in 1640 (for BEL-7402) or Dulbecco's Modified Eagle Medium (for Human Embryonic Kidney Cells 293T and Huh7) medium (Life Technologies, USA) supplemented with 10% (v/v) fetal bovine serum (Life Technologies) and antibiotics (100 IU/mL penicillin and 100 μg/mL streptomycin).

### Isobaric tags for relative and absolute quantitation (iTRAQ)

#### Protein extraction and quantification

Huh7 cells (including *ATP7B*-knockdown and negative control cell lines) were incubated with 100 μM CuSO_4_ for 0, 24, and 48 hrs. Proteins were extracted using lysis buffer (0.1 M Tris/HCl buffer containing 7 M urea, 2 M thiourea, 0.1% phenylmethylsulfonyl fluoride, and 65 mM dithiothreitol, pH 8.0), and sample concentration was determined using a Bradford protein assay kit (Thermo, USA); 50 μg of protein were used for each sample. The detailed protocol for isobaric tags for relative and absolute quantitation (iTRAQ) analysis has been published previously.^26^

#### Liquid chromatography and tandem mass spectrometry analysis

Liquid chromatography and tandem mass spectrometry (LC–MS/MS) was performed by the National Center for Protein Sciences (Beijing). In brief, protein samples were precipitated with acetone-trichloroacetic acid and trypsin digested to generate proteolytic peptides. Proteolytic peptides were then labeled with iTRAQ reagents. The iTRAQ labels 113–118 were used to separately label the samples at various time points, and labeled samples were pooled before further analysis. The combined peptide mixtures were analysed using LC–MS/MS for both identification and quantification. The “abundances” indicated the quantification of the protein, and the fold change was calculated by comparing the abundances of different samples.

#### Bioinformatic analysis

The lists of transcription factors, splicing factors, and ion transporters were obtained from the Human Protein Atlas (http://www.proteinatlas.org), RBPmap (http://rbpmap.technion.ac.il/index_DEV.html), and HitPredict (http://www.hitpredict.org/) databases, respectively. Gene ontology (biological process) and Kyoto Encyclopedia of Genes and Genomes pathway enrichment analysis were performed using DAVID (https://david.ncifcrf.gov/).

### The wild-type and p.S29L (c.86C > T) mutant CTR2-expressed cell lines

Wild-type (WT) and mutant (Mut) human *CTR2* cDNA sequences were chemically synthesized by Tsingke Biotechnology Co., Ltd (Beijing, China) and cloned into the pCDH–EF1–MCS–T2A–copGFP (pCDH) vector (MiaoLing Plasmid Platform, China); HEK-293T (National Collection of Authenticated Cell Cultures) lentivirus packaging cell lines were used to generate virus particles. Following co-transfection of pCDH, psPAX2, and pMD2.G plasmids (MiaoLing Plasmid Platform) in to 293T cells, replication-incompetent virions (CTR2^WT^ and CTR2^Mut^) were released into the culture medium supernatant, and the supernatant were collected after 48 and 72 hrs. The procedure was referred to the instructions provided by addgene (https://www.addgene.org/protocols/lentivirus-production/).

BEL-7402 and Huh7 cells (National Collection of Authenticated Cell Cultures) were co-incubated with CTR2^WT^ and CTR2^Mut^ lentivirus for 72 hrs. The copGFP^+^ cells were selected using a flow cytometer, and were cultured as stable WT and Mut cell lines (BEL-7402^CTR2-WT/Mut^ and Huh7^CTR2-WT/Mut^).

#### CCK-8 assays

The cells were seeded onto 96-well plates, 5 × 10^3^ cells/well, in 80 μL of 1640 (BEL-7402 cells) or DMEM (Huh7 cells), and supplemented with of CuSO_4_ and/or D(−)-penicillamine (D-PEN) (Sigma, USA). After 0, 24, 48, and 72 hrs, 10 μL of CCK-8 solution (Cell Counting Kit, Yeasen Biotechnology, China) dissolved in 90 μl of 1640 or DMEM was added to each well. The plates were incubated for 2 hrs at 37°C, and the absorbance was measured at 450 nm.

#### Flow cytometry

Huh7^CTR2-Con/WT/Mut^ cells were seeded onto 6-well plates overnight. The cells were treated with 200 μM of CuSO_4_ for 24 hrs, then 1mM D-PEN was added in the freshly changed culture medium for further incubation. After 24 hrs, the cells were collected and washed twice with PBS. The cells were stained with Annexin V-PE/7-AAD (PE Annexin V Apoptosis Detection Kit I, BD Biosciences, USA) on ice for 15 min, flow cytometric analysis was conducted, and apoptotic fractions were determined.

### Quantification of copper ion concentration

Huh7^CTR2-Con/WT/Mut^ cells were seeded onto 100 mm cell culture dishes, and supplemented with 200 μM of CuSO_4_ and/or 1mM D-PEN. After 24 hrs, the cells were harvested and washed twice by 2 mL PBS. The cell number was calculated by TC10 automated cell counter (Bio-Rad, USA). The quantification of copper ion concentration was measured using QuantiChrom^TM^ Copper Assay Kit (BioAssay Systems, USA).

### Statistical analysis

All *in vitro* experiments were repeated at least three times. Two-tailed unpaired Student's *t*-tests and one-way ANOVA were used to evaluate differences between groups. GraphPad Prism 7 and MedCalc were used for statistical analyses. All quantitative data are presented as mean ± SD, and *P* < 0.05 was considered statistically significant.

## Results

### Genetic and clinical profiles of the WD patients

Sanger sequencing was performed in our WD patient cohort recruited from the CR-GMLD Group. Single or no *ATP7B* mutations were detected in 10 of the patients. These patients were selected for further WES. The pathogenicity of the variants was analysed using prediction tools including Polyphen-2, MutationTaster, and SIFT (Table 1). Sanger sequencing identified seven different pathogenic variants in CDS regions, including the most common *ATP7B* variant p.R778L (3 in 10 patients), five other missense mutations (p.A874V, p.T935M, p.H1019P, p.V1106I, and p.N1270S), and one small In/Del mutation (p.F699-). In patient S3, no pathogenic variant was detected. SNPs were identified in patient S2 (p.S406A, p.V456L, p.K832R, and p.R952K), S3 (p.S406A, p.V456L, p.K832R, and p.R952K), S5 (p.S406A and p.V456L), and S7 (p.S406A, p.V456L, p.K832R, p.R952K, and p.V1140A).

**Table 1.** *ATP7B* pathogenic variants detected in 10 patients with Wilson disease

			Pathogenicity		
Patient	Sanger sequencing	Whole-exome sequencing	Polyphen-2	MutationTaster	SIFT
S1	p.R778L	p.R778L	Probably damaging	Disease causing	Damaging
S2	p.V1106I	p.V1106I	Probably damaging	Disease causing	Tolerable
S3	-				
S4	p.F699-	p.F699-		Disease causing	
S5	p.H1019P	p.H1019P	Probably damaging	Disease causing	Damaging
		Large fragment deletions (chr13:52 544 737-52 548 212 and chr13:52 549 161-52 585 503)			
S6	p.R778L	p.R778L	Probably damaging	Disease causing	Damaging
S7	p.T935M	p.T935M	Probably damaging	Disease causing	Damaging
S8	p.N1270S	p.N1270S	Probably damaging	Disease causing	Damaging
S9	p.A874V	p.A874V	Probably damaging	Disease causing	Damaging
S10	p.R778L	p.R778L	Probably damaging	Disease causing	Damaging

The clinical profiles of the 10 WD patients, including six males and four females from 10 unrelated families, are summarized in Table 2. In general, these patients had a high Leipzig score (≥4) and a low CP level (<0.2 g/L). Seven of 10 patients were hepatic phenotypes, including abnormal liver biochemistry and cirrhosis, and a hepatic and neurological mixed presentation was observed in the remaining three patients. In addition to the hepatic phenotypes, typical symptoms included tremors and involuntary movements.

**Table 2.** The clinical profiles of the 10 Wilson disease patients

Patient	Clinical presentation	Age	Gender	Lepziq score	Serum copper (μmol/L)	Ceruloplasmin (g/L)	Urine copper (μg/24 hrs)	K-F rings	Clinical symptoms	Abdominal ultrasound	Brain MRI
S1	Hepatic	27	M	4	Not available	0.082	Not available	Negative	Anorexia and retching for >1 month, transaminase elevated for 1 week	Cirrhosis, splenomegaly	Not available
S2	Hepatic	44	M	4	Not available	<0.01	Not available	Not available	Abnormal liver function	Not available	Not available
S3	Mixed	31	M	5	Not available	0.13	400.1	Not available	The patient had the onset of seizures at age 7 years and was diagnosed with epilepsy. The patient was found to have liver cirrhosis, splenomegaly, and ascites at age 18 years.	Cirrhosis, splenomegaly, ascites, cavernous transformation of portal vein, widened spleen vein, abnormal nodules in hepatic segments	Not available
S4	Hepatic	4	W	4	Not available	0.04	88.9	Negative	Physical examination revealed abnormal liver function, without jaundice and edema.	No abnormality found	Abnormalities of the body of bilateral ventricles
S5	Mixed	14	W	5	7.3	0.05	Not available	Positive	Dysarthria, abnormal gait, abdominal distension, and loss of appetite for 1 month. Physical examination shows splenomegaly.	Cirrhosis, splenomegaly	Abnormalities of the body of bilateral ventricles
S6	Hepatic	15	W	7	Not available	<0.01	649.4	Positive	Lower limb edema, rash, facial edema for 1 month	Multiple hyperechoic nodules and diffuse disease and multiple hyperechoic nodules of the liver	No abnormality found
S7	Hepatic	13	M	5	Not available	0.05	196	Negative	Abnormal liver function, accompanied by occasional abdominal discomfort	No abnormality found	No abnormality found
S8	Hepatic	22	M	4	11.4	0.14	145.2	Not available	Physical examination revealed abnormal liver function without any symptoms	No abnormality found	Left temporal lobe cyst
S9	Mixed	36	M	7	Not available	0.02	2160	Positive	Involuntary tremor of head for 7 years	Cirrhosis, splenomegaly	Multiple ischemic changes in white matter, bilateral ethmoid sinusitis
S10	Hepatic	4	W	5	Not available	<0.01	116	Not available	Physical examination revealed abnormal liver function, the penicillamine challenge test (+).	Not available	Not available

The WES was performed by MyGenostics Inc. (Beijing, China). In addition of the pathogenic variants we detected by Sanger sequencing, in patient S5, we identified large fragment deletions (chr13:52 544 737-52 548 212 and chr13:52 549 161-52 585 503) in one of the *ATP7B* alleles. These mutations were identified by WES, but failed to be detected by Sanger sequencing (Table 1). Pedigree analysis revealed that the patient was a compound heterozygote.

### Discovering potential modifier genes in nine WD patients

We analysed the WES data of the remaining nine patients with WD. Variants with a frequency <0.01 in 1000 Genome, ESP6500, ExAC_ALL, and ExAC_EAS databases were selected for further analysis. Multiple function prediction software, including Polyphen-2, MutationTaster, and SIFT, were used to predict the pathogenicity of nonsynonymous variants with uncertain clinical significance. Five hundred and thirteen candidate genes, of which the genetic variants present in at least two patients, were finally identified (Supplemental File S1). These candidate genes included 23 transcription factors and two splicing factors (Table 3), and 14 metal ion transport protein encoding genes [including *SLC31A2* (encoding CTR2); Table 3]. These transporters might have synergistic or competitive effects on copper transport. However, no metal ion metabolism-related biological processes were significantly enriched (Supplementary Fig. S1). No pathogenicity variants were identified in those previously reported modifier genes, including *ATOX1, COMMD1, XIAP*, and *HFE*.

**Table 3.** Genetic variants in transcription factors, splicing factors, and metal ion transporters

Gene	Nucleotide Changes	Amino Acid Changes	Gene Type	ExAC EAS/ALL	gnomAD EAS/ALL	dbSNP	PolyPhen-2	Mutation Taster	SIFT	InterVar	Disease (OMIM)
Transcription factors											
AR	c.1369_1377 delGGCGGCGGC	p.457_459 delGGG	het	-/-	0.0366/0.0378					Uncertain	313 200|300 068|312 300|300 633|176 807
AR	c.1369_1374 delGGCGGC	p.457_458 delGG	hom	-/-	0.0026/0.0354					Uncertain	313 200|300 068|312 300|300 633|176 807
FOXI2	c.182C > A	p.P61Q	het	-/-	0.0074/0.0004	rs118034115	Benign	Polymorphism	Tolerated	Uncertain	#N/A
FOXK2	c.1899G > C	p.K633N	het	0.0099/0.0007	0.008/0.0005	rs117690256	Possibly_damaging	Disease_causing	Damaging	Uncertain	#N/A
FOXK2	c.500C > T	p.A167V	het	0.0135/0.001	0.0167/0.0009	rs150523977	Benign	Disease_causing	Tolerated	Uncertain	#N/A
FOXO3	c.1141dupG	p.L382Afs*2	het	-/-	0/0	rs34133353				Uncertain	#N/A
FOXO3	c.184G > A	p.D62N	het	0.0043/0.0022	0.0043/0.0002	rs532258926	Benign	Disease_causing	Damaging	Uncertain	#N/A
GLI2	c.3623G > T	p.R1208L	het	-/-	-/-		Benign	Polymorphism	Tolerated	Uncertain	615 849|610 829
KLF4	c.1100-7C > A		het	0.0115/0.0011	0.0123/0.0006	rs188071068				Uncertain	#N/A
MGA	c.9112C > T	p.R3038W	het	0.0044/0.0004	0.0037/0.0002	rs117068470	Possibly_damaging	Disease_causing	Damaging	Uncertain	#N/A
NCOR2	c.1529_1531 delAGC	p.510delQ	het	0.0031/0.0036	-/-	rs753830534				Uncertain	#N/A
NCOR2	c.2033A > C	p.N678T	het	-/-	-/-		Possibly_damaging	Disease_causing	Tolerated	Uncertain	#N/A
NFE2L3	c.166G > C	p.A56P	het	-/-	0.0012/0.00 006 525	rs748882173	Possibly_damaging	Polymorphism	Damaging	Uncertain	#N/A
NR1H2	c.695C > T	p.A232V	het	0.0002/0.0002	-/-	rs201627190	Benign	Disease_causing	Tolerated	Uncertain	#N/A
NR1H2	c.211C > T	p.R71C	het	0.0008/0.00 005 957	0/0.00 003 234	rs756403846	Benign	Disease_causing	Damaging	Uncertain	#N/A
PDX1	c.127_129 delCCG	p.43delP	het	-/0.0032	0.0006/0.00 003 243	rs752633548				Uncertain	606 392|260 370|125 853
RUNX2	c.232_234 delGCG	p.78delA	het	-/0.0006	0/0.00 003 362	rs756546787				Uncertain	119 600|156 510
TRPS1	c.592A > G	p.K198E	het	-/0.000 008 282	-/-	rs754340524	Benign	Polymorphism	Damaging	Uncertain	190 350|190 351
ZC3H6	c.871C > G	p.Q291E	het	0.0052/0.0003	0.0065/0.0004	rs375792649	Benign	Disease_causing	Damaging	Uncertain	#N/A
ZFHX2	c.1108G > T	p.A370S	het	-/-	-/-			Polymorphism	Damaging	Uncertain	147 430|613 750
ZNF23	c.583C > A	p.P195T	het	0.0054/0.0004	0.0068/0.0004	rs76530144	Possibly_damaging	Polymorphism	Damaging	Likely_benign	#N/A
ZNF333	c.452T > C	p.I151T	het	0.0025/0.0002	0.0018/0.00 009 684	rs141998898	Benign	Polymorphism	Tolerated	Uncertain	#N/A
ZNF438	c.590G > A	p.G197E	het	0.0074/0.0006	0.0062/0.0003	rs148252990	Benign	Polymorphism	Tolerated	Likely_benign	#N/A
ZNF469	c.11053C > T	p.R3685C	het	0.0025/0.00 006 775	0.0043/0.0003	rs748126365	Benign	Polymorphism	Damaging	Uncertain	229 200
ZNF469	c.1663G > A	p.D555N	het	0.0047/0.0002	0.0025/0.0001	rs749179728	Possibly_damaging	Polymorphism	Damaging	Uncertain	229 200
ZNF644	c.2707G > A	p.G903R	het	-/-	-/-		Probably_damaging	Disease_causing	Damaging	Uncertain	614 167
ZNF655	c.413T > C	p.F138S	het	0.0156/0.0011	0.0167/0.0009	rs149822831	Possibly_damaging	Disease_causing	Damaging	Uncertain	#N/A
ZNF669	c.89G > T	p.R30L	het	0.0157/0.0011	0.0111/0.0006	rs201216518	Benign	Polymorphism	Tolerated	Likely_benign	#N/A
ZSCAN25	c.307C > T	p.R103W	het	0.0035/0.0015	0.0055/0.0014	rs145815306	Benign	Polymorphism	Damaging	Uncertain	#N/A
Splicing factors											
ANKHD1	c.74C > T	p.P25L	het	0.0013/0.0001	0.0018/0.00 009 718	rs557072130	Benign	Disease_causing	Damaging	Uncertain	#N/A
RBM45	c.652A > G	p.R218G	het	0.0054/0.0004	0.008/0.0004	rs138537367	Benign	Disease_causing	Tolerated	Uncertain	#N/A
Metal ion transporters											
ABCA10	c.3103T > C	p.S835P	het	-/-	-/-		Probably_damaging	Disease_causing	Damaging	Uncertain	#N/A
ABCA10	c.2260C > T	p.R754X	het	0.0071/0.0005	0.0025/0.0001	rs190646470		Disease_causing		Uncertain	#N/A
ATP6V1C2	c.923G > A	p.G308E	het	0.0047/0.0004	0.0062/0.0003	rs117054621	Benign	Disease_causing	Damaging	Uncertain	#N/A
CACNA1F	c.3094G > A	p.E1032K	het	-/0.00 006 846	0/0.00 004 745	rs200976011	Possibly_damaging	Disease_causing	Tolerated	Uncertain	300 071|300 476|300 600
KCNG4	c.778T > C	p.Y260H	het	0.0076/0.0006	0.0105/0.0005	rs117787002	Benign	Disease_causing	Tolerated	Uncertain	#N/A
KCNJ12	c.139G > A	p.V47I	het	0.0075/0.0011	0.0032/0.0006	rs201403828	Possibly_damaging	Disease_causing	Tolerated	Uncertain	#N/A
SLCO1A2	c.1681A > G	p.I561V	het	0.0106/0.0008	0.0117/0.0006	rs116587731	Probably_damaging	Disease_causing	Damaging	Uncertain	#N/A
SLCO1A2	c.1736dupG	p.T580Nfs*5	het	0.0007/0.00 005 773	0.0006/0.00 003 228	rs3830207				Uncertain	#N/A
SLC26A9	c.2621delC	p.P874Hfs*19	het	0.0069/0.0006	0.0111/0.0006	rs536446020				Uncertain	#N/A
SLC31A2	c.86C > T	p.S29L	het	0.0022/0.0002	0.0024/0.0002	rs200288219	Probably_damaging	Disease_causing	Damaging	Uncertain	#N/A
SLC34A3	c.473C > T	p.P158L	het	0.0002/0.00 001 754	-/-	rs771246525	Probably_damaging	Disease_causing	Damaging	Uncertain	241 530
SLC39A4	c.592G > A	p.A198T	het	0.0016/0.0002	0.0012/0.0001	rs781818806	Benign	Polymorphism	Tolerated	Uncertain	201 100
SLC5A3	c.1415A > G	p.Y472C	het	0.0074/0.0006	0.0099/0.0005	rs117638044	Benign	Disease_causing	Damaging	Uncertain	#N/A
SLC7A2	c.1301A > G	p.Y434C	het	0.0017/0.0002	-/-	rs201373242	Probably_damaging	Disease_causing	Damaging	Uncertain	#N/A

dbSNP: Database for single nucleotide polymorphisms; EAS: East Asian.

**Figure 1.**
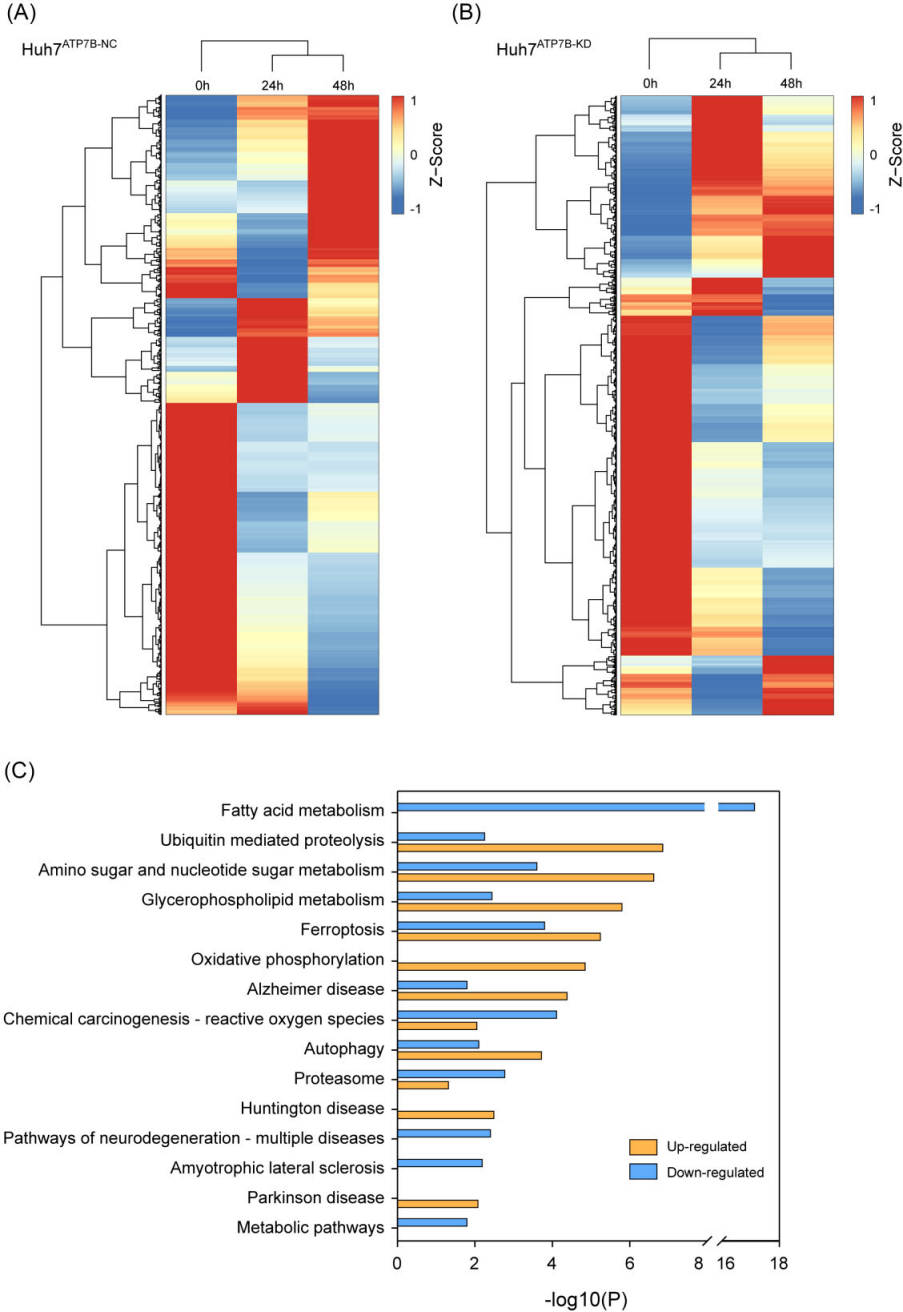
The protein expression changes in Cu-stimulated cell lines. (A) and (B) The protein expression patterns were quite similar after Cu stimulation at various time points in Huh7^ATP7B-NC^ and Huh7^ATP7B-KD^ cells. (C) KEGG analysis revealed that the metabolism-related pathways were highly enriched by those down-regulated proteins, while the up-regulated proteins were mainly functioning in nervous system disease–related pathways.

We next examined which proteins might be involved in copper transfer and metabolism processes, or affected by copper stimulation. The iTRAQ was performed to identify proteins differentially expressed between normal and Cu-stimulated cell lines. We generated the *ATP7B*-knockdown stable Huh7 cell lines by lentivirus. The Huh7^ATP7B-NC^ and Huh7^ATP7B-KD^ cells were co-incubated with 200 μM of CuSO_4_ for 0, 24, and 48 hrs, then the cells were harvested and protein samples were digested to generate proteolytic peptides. The proteolytic peptides were labeled with iTRAQ reagents. Protein expression patterns were quite similar in Huh7^ATP7B-NC^ and Huh7^ATP7B-KD^ cell lines (Fig. 1A and B). Proteins with 1.2-fold increase or 0.83-fold decrease and *P*-value (<0.05)^27–29^ were considered differentially expressed. Up-regulated (*n* = 363) and down-regulated (*n* = 949) proteins were identified (Supplemental File S2). KEGG analysis revealed that the metabolism-related pathways were highly enriched by those down-regulated proteins; while the up-regulated proteins were mainly functioning in nervous system disease–related pathways, including Alzheimer disease, Huntington disease, and Parkinson disease–related pathways (Fig. 1C), which consistent with the typical clinical features of WD (liver abnormalities and neurological symptoms).

Thirteen genes/proteins were identified in both WES and iTRAQ data, including TTN, LAMA5, PRSS1, PLEKHA7, CDC27, DYNC2H1, FBLIM1, FDXR, MRTFB, NLRP13, PISD, NOP53, and PIH1D1 (Table 4). Only PLEKHA7 was reported to regulate the localization and function of ATP7A to promote copper extrusion in elevated copper through WW–PLEKHAs (PLEKHA5, PLEKHA6, PLEKHA7)–PDZD11 complexes^30,31^, no reports published to date indicate that the rest proteins are involved in copper ion intercellular transportation or metabolism processes. The molecular basis through which these proteins may be involved in WD requires further investigation. For some of the potential copper responsive proteins reported previously, the information from iTRAQ, WES, and literature search, were combined together in Table 5.

**Table 4.** Genetic variants in 13 candidate genes/proteins that identified in both whole-exome sequencing and isobaric tags for relative and absolute quantitation data

Gene	Nucleotide Changes	Amino Acid changes	Gene Type	ExAC EAS/ALL	gnomAD EAS/ALL	dbsnp150	PolyPhen-2	Mutation Taster	SIFT	InterVar	Disease (OMIM)
TTN	c.38708-4T > C	Splicing	het	0.0083/0.0077	0.0049/0.0057	rs200819643				Uncertain	#N/A
TTN	c.22544T > C	p.I7515T	het	0.0006/0.00 004 208	0.0003/0.00 003 672	rs199918663	Benign	Disease_causing	Damaging	Uncertain	#N/A
TTN	c.44174G > A	p.R14725H	het	0.019/0.0014	0.0164/0.0012	rs55677134	Benign	Disease_causing	Tolerated	Likely_benign	#N/A
LAMA5	c.7145T > G	p.M2382R	het	0.0001/0.000 008 417	0.0001/0.000 008 215	rs774236266	Probably_damaging	Disease_causing	Tolerated	Uncertain	#N/A
PRSS1	c.623G > C	p.G208A	het	0.0091/0.0007	0.0092/0.0007	rs189270875	Probably_damaging	Disease_causing	Damaging	Uncertain	Pancreatitis, chronic
PRSS1	c.637G > A	p.V213I	het	0.0079/0.0032	0.0001/0.00 001 641	rs200902389	Benign	Polymorphism	Tolerated	Likely_benign	#N/A
PRSS1	c.652G > T	p.D218Y	het	0.0002/0.00 007 415	0.0003/0.00 004 471	rs574391339	Benign	Polymorphism	Tolerated	Uncertain	#N/A
CDC27	c.628_630delATT	p.210delI	het	-/-	-/-					Uncertain	#N/A
CDC27	c.477_478insCCCTTTGAATCATGATGTGAAATAGGGGG	p.E160_K161delinsPFESX	het	-/-	-/-					Uncertain	#N/A
CDC27	c.1750A > G	p.S584G	het	-/-	0/0	rs62075623	Possibly_damaging	Disease_causing	Damaging	Uncertain	#N/A
CDC27	c.1456A > C	p.N486H	het	0.0009/0.0008	0.0005/0.0002	rs62077261	Possibly_damaging	Disease_causing	Damaging	Uncertain	#N/A
CDC27	c.1424T > G	p.M475R	het	-/-	0.0002/0.0001	rs74496366	Benign	Disease_causing	Damaging	Uncertain	#N/A
CDC27	c.627A > C	p.T209T	het	-/-	-/-	rs144985864				Likely_benign	#N/A
DYNC2H1	c.2909A > G	p.N970S	het	-/-	-/-		Benign	Disease_causing	Tolerated	Uncertain	#N/A
FBLIM1	c.1092_1093del	p.T365Efs*12	het	-/-	-/-					Uncertain	#N/A
FDXR	c.11G > A	p.R4H	het	-/-	-/-		Probably_damaging	Disease_causing	Damaging	Uncertain	#N/A
FDXR	c.1252G > A	p.G418S	het	0.0031/0.0008	0.002/0.0009	rs141101303	Benign	Disease_causing	Tolerated	Uncertain	#N/A
MRTFB	c.683C > A	p.T228N	het	0.0001/0.000 008 273	0.0002/0.00 001 231	rs747644210	Benign	Disease_causing	Tolerated	Uncertain	#N/A
NLRP13	c.364C > G	p.L122V	het	0.0042/0.0003	0.0029/0.0002	rs141703645	Benign	Polymorphism	Tolerated	Uncertain	#N/A
NOP53	c.1309A > G	p.I437V	het	0.0046/0.0003	0.0041/0.0003	rs199555316	Benign	Disease_causing	Tolerated	Uncertain	#N/A
PIH1D1	c.727C > T	p.R243C	het	0.007/0.0024	0.0079/0.0023	rs149419497	Possibly_damaging	Disease_causing	Tolerated	Uncertain	#N/A
PISD	c.187G > A	p.V63I	het	0.0101/0.0008	0.0102/0.0007	rs183406976	Benign	Disease_causing	Tolerated	Likely_benign	#N/A
PISD	c.667T > G	p.S223A	het	-/-	-/-		Benign	Disease_causing	Tolerated	Uncertain	#N/A
PLEKHA7	c.2585C > T	p.P862L	het	0.0046/0.0004	0.0055/0.0003	rs201660932	Benign	Disease_causing	Tolerated	Uncertain	#N/A
PLEKHA7	c.1037G > A	p.R346H	het	0.004/0.0003	0.0018/0.00 009 688	rs78500033	Benign	Polymorphism	Tolerated	Uncertain	#N/A
PLEKHA7	c.157C > T	p.R53C	het	0.0131/0.001	0.0105/0.0006	rs201642255	Probably_damaging	Disease_causing	Damaging	Uncertain	#N/A

**Table 5.** Potential copper responsive proteins in WES and iTRAQ data

Potential copper responsive proteins	Mutation (WES)	Protein expression (iTRAQ)	Literature (PMID)
AP1S1		<1.2-fold change	27 603 756
ABCA12			546 910
ATOX1		Down-regulation	12 539 964
ATP11A		Down-regulation	27 603 756
ATP6AP1		Up-regulation	27 603 756
ATP7A			23 064 757
CCS		<1.2-fold change	12 539 964
Ceruloplasmin			11 461 924
CLC7			20 530 571
COMMD6		Up-regulation	27 603 756
COMMD9		Up-regulation	27 603 756
COX1			16 760 263
COX11			25 926 683
COX17		<1.2-fold change	18 458 339
COX2			16 760 263
DMT1			26 294 671
GRX1			20 566 629
P2RX4		Down-regulation	27 603 756
SCO1		Down-regulation	18 458 339
SCO2		Down-regulation	24 403 053
SLC12A2		Down-regulation	27 603 756
SLC22A18		Up-regulation	27 603 756
SLC25A20		Down-regulation	27 603 756
SLC31A1 (CTR1)		Down-regulation	11 734 551
SLC31A2 (CTR2)	c.86C > T, p.S29L		26 342 034
SLC33A1		Down-regulation	27 603 756
SLC38A1		Down-regulation	27 603 756
SLC39A5			15 322 118
SLC7A2	c.1301A > G, p.Y434C	<1.2-fold change	27 603 756
SOD1		<1.2-fold change	12 539 964
STEAP			16 609 065

iTRAQ: isobaric tags for relative and absolute quantitation.

### The CTR2 p.S29L variant promotes the Cu-induced apoptosis in BEL-7402 and Huh7 cells

**Figure 2.**
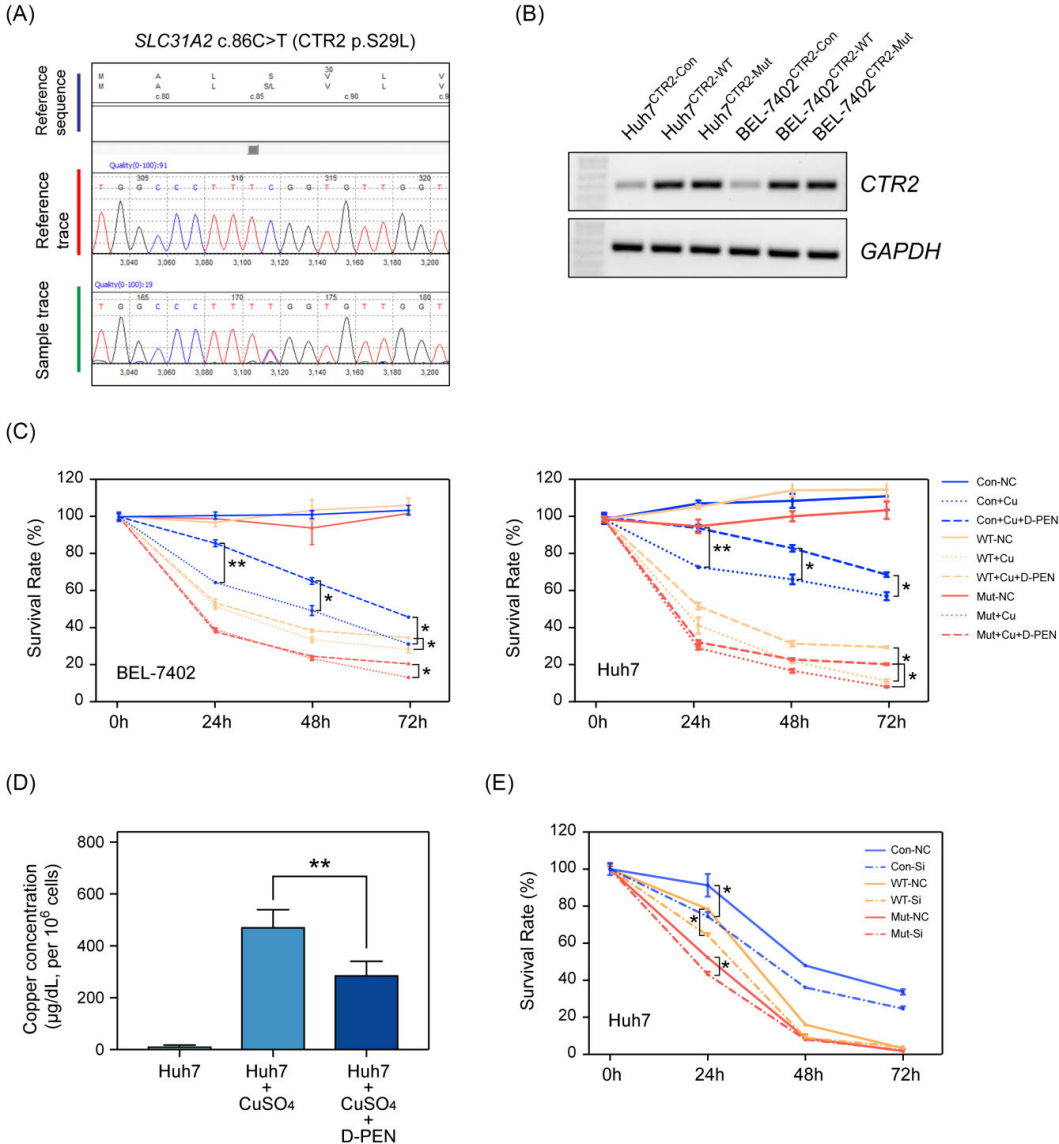
The pathogenicity of CTR2 p.S29L variant. (A) Sanger sequencing confirmed the variant of *SLC31A2* c.86C > T (CTR2 p.S29L). (B) The RNA expression of wild-type (WT) and mutant CTR2 in Huh7 and BEL-7402 cells. (C) Overexpression of mutant CTR2 caused a greater amount of Cu-induced apoptosis than their WT counterparts, and D(−)-penicillamine (D-PEN) reversed the phenotype. (D) CuSO_4_ incubation caused an enhanced uptake of the copper ion in Huh7 cells, and D-PEN was able to decrease the intracellular copper concentration. The Cu concentration has been normalized to cell number (per 10^6^ cells). (E) The mutant CTR2 caused significant reduced survival rate in Huh7^ATP7B-KD^ cells. *, *P* < 0.05 and **, *P* < 0.01.

CTR2 is an influx Cu transporter in mammalian cells. Because of the low affinity to copper ion, the function of CTR2 is still not fully understood. Our WES analysis identified a c.86C > T missense mutation in exon 3 of the *SLC31A2* gene. An additional 191 patients with WD and 109 healthy donors were recruited for detecting the *SLC31A2* c.86C > T (CTR2 p.S29L) variant using Sanger sequencing (Fig. 2A). Our results showed a much higher *SLC31A2* c.86C > T frequency in our WD cohort (6/191, 0.0314) than in East Asian (0.0024 in gnomAD database, *P* < 0.0001, test for one proportion) or worldwide (0.0002 in gnomAD database, *P* < 0.0001, test for one proportion) populations. Furthermore, none of the 109 healthy donors carried the *SLC31A2* c.86C > T variant; despite this, no significant difference was observed (*P* = 0.090, Fisher exact probability), probably because of the extremely low frequency even in WD patients and the limited number of healthy donors. Multiple function prediction software, including Polyphen-2, MutationTaster, and SIFT, indicated that this single-base substitution mutation has strong pathogenicity (Table 3).

We preliminarily assessed the pathogenicity of CTR2 p.S29L in cultured cell lines. Considering the low expression of endogenous CTR2 in BEL-7402 and Huh7 cells, we constructed BEL-7402^CTR2-WT/Mut^ and Huh7^CTR2-WT/Mut^ stable cell lines by directly overexpression of CTR2-WT/Mut genes using lentivirus (Fig. 2B). The cells were seeded onto 96-well plates and co-incubated with 200 μM of CuSO_4_ for 24 hrs, then 1 mM  D-PEN was added in the freshly changed culture medium for further incubation. CTR2^S29L^ caused a greater amount of Cu-induced apoptosis in both BEL-7402^CTR2-Mut^ and Huh7^CTR2-Mut^ cells after 24 hrs (Fig. 2C). The Cu-induced phenotype could be rescued by 1 mM of D-PEN at 48 and 72 hrs, in cells with negative control, WT, or mutant CTR2.

**Figure 3.**
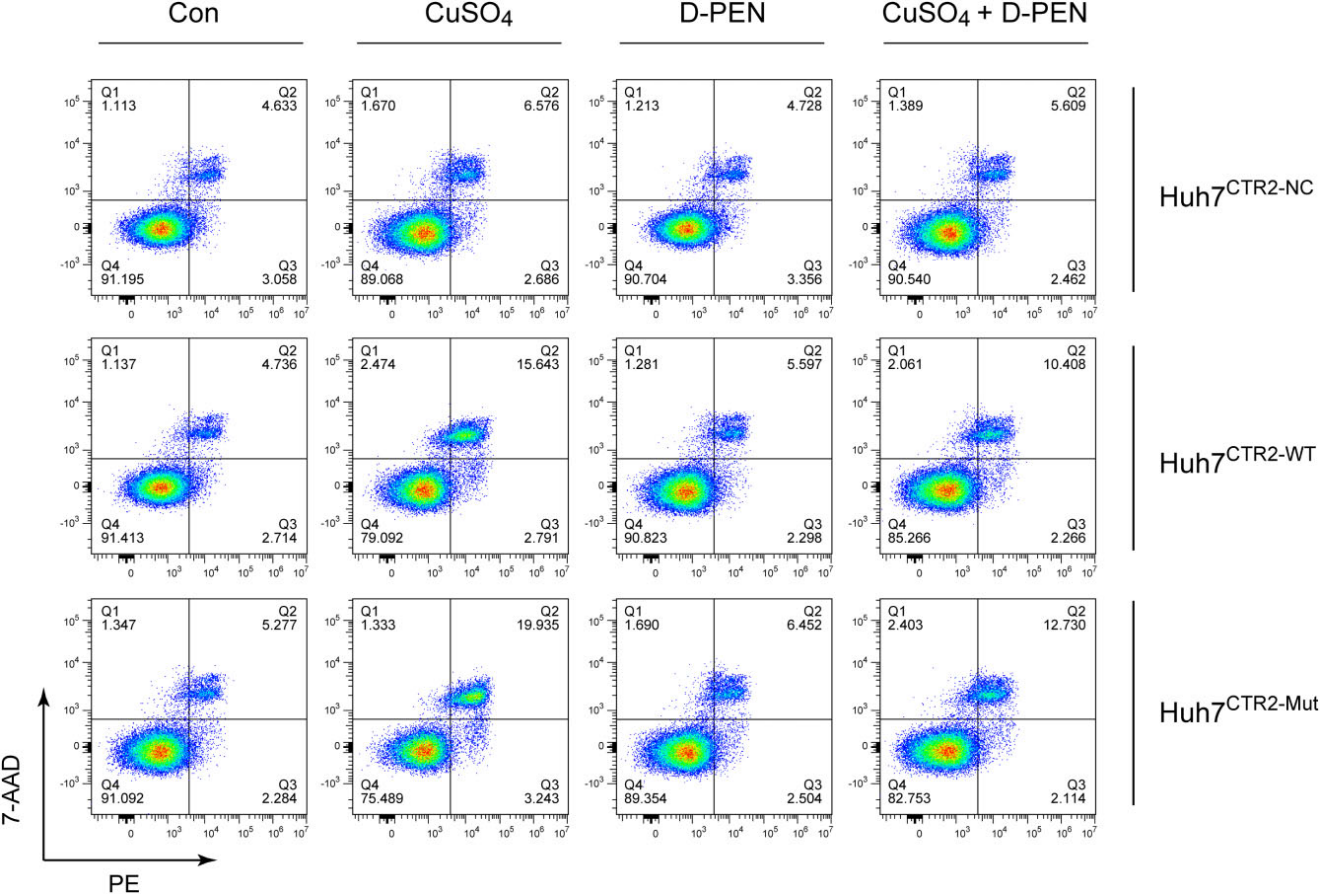
The flow cytometry indicated the apoptotic proportion of the Cu-stimulated cells. Copper stimulation induced more apoptosis in cells expressing wild-type (WT) and Mut CTR2. 15.643 and 19.935% of cells were apoptotic in Cu-stimulated Huh7^CTR2-WT^ and Huh7^CTR2-Mut^ cells, respectively, and 1 mM of D(−)-penicillamine (D-PEN) reduced the proportion to 10.408 and 12.730%.

The apoptosis of the Cu-stimulated cells was detected by flow cytometry. After co-incubation with 200 μM of CuSO_4_ for 24 hrs, 19.935% of cells were apoptotic in Cu-stimulated Huh7^CTR2-Mut^ cells (15.643% in Huh7^CTR2-WT^ cells), and 1mM of D-PEN reduced the proportion to 12.730% (10.408% in Huh7^CTR2-WT^ cells) (Fig. 3). The increased Cu-induced apoptosis might be due to the enhanced uptake of the copper ion. We further assessed the intracellular Cu concentration in Huh7 cells, with CuSO_4_ and/or D-PEN stimulation. CuSO_4_ incubation caused an enhanced uptake of the copper ion in Huh7 cells, and D-PEN was able to decrease the intracellular copper concentration (Fig. 2D).

We further used siRNA pools to knock down *ATP7B* gene in Huh7 cells that express NC, WT, and mutant CTR2. The knockdown of *ATP7B* gene caused varying degrees of apoptosis in Cu-stimulated Huh7 cells, including the cells that with only endogenous CTR2. Although the cells with WT CTR2 also revealed enhanced apoptosis than negative controls, the cells with mutant CTR2 showed significant reduced survival rate compared with their WT counterparts at 24 hrs (Fig. 2E).

## Discussion

Although with only single or no heterozygous mutations, some of the patients may still exhibit severe WD manifestations, they do not obviously differ from those with pathogenic homozygous or compound heterozygous mutations. In the current study, all of the 21 exons and at least 50 bp of intronic flanking sequences of the *ATP7B* gene were amplified by Sanger sequencing, and the multiplex ligation-dependent probe amplification test was performed to make sure that there was no large deletions in the promotor region of the *ATP7B* gene (data not shown). To our knowledge, no pathogenic mutations in deep intronic sequences that cause WD have been reported. Thus, the phenotypic variability and incomplete penetrance observed in WD suggests the involvement of modifier genes. To date, rare variants in several genes have been associated with WD, and a recent report using WES showed an increased and reduced risk of neurological presentation by variants in *ESD* and *INO80* genes, respectively.^32^ Furthermore, rare *APOE* and *MBD6* variants are associated with lower risk of early onset WD.^32^

Although the search for WD modifier genes is difficult, it is worth pursuing. The identification of these modifiers may provide a better understanding of the biological pathways that WD indirectly affects, and could also direct therapy possibilities for patients with WD.^33^ Several strategies have been proved effective in identifying modifiers, including linkage analysis, association studies, biology-driven approaches, and systematic screens.^33^ WES captures and sequences the entire exome; in the human genome, WES covers approximately 22 000 protein-coding genes.^34^ This approach can also be used to capture low-frequency copy number variations or complicated In/Del mutations.^34^ In this study, WES identified two large fragments deleted in the *ATP7B* gene in one of our patients, which was not detected by Sanger sequencing. However, a WES strategy might also be disappointing when searching for potential modifier genes, especially where limited samples are available. Here, we identified 513 potential modifiers with genetic variants in at least two patients, but no metal ion metabolism-related biological processes were significantly enriched. The genes with potential mutations within the 3′ or 5′ UTRs (untranslated regions) which encode copper-binding proteins, may serve as modifier genes. The targeted next-generation sequencing enables rapid identification of genetic variations of a series of gene sets, both in exonic, intronic, and UTR regions. Thus, using the targeted next-generation sequencing for rare pathogenic mutations of copper metabolism-related genes will be helpful in searching the potential modifier genes of WD. Therefore, it may be wiser to focus on a more limited number of genes, the so-called candidate genes, on the basis of biological knowledge or molecular experiments.

To date, only a limited number of the variants identified in “modifier genes” have been confirmed by molecular experiments or other research. Using WES and iTRAQ approaches, we identified 13 candidate modifier genes, rare variants of which were supposed to be highly involved in the WD phenotype. However, the function annotations in the UniProt database (https://www.uniprot.org/) did not indicate a relationship between those candidates and WD phenotype. To our knowledge, so far, only PLEKHA7 was reported to regulate the localization and function of ATP7A to promote copper extrusion in elevated copper through WW–PLEKHAs (PLEKHA5, PLEKHA6, PLEKHA7)–PDZD11 complexes^30,31^; no reports published to date indicate that the rest of the proteins are involved in copper ion intercellular transportation or metabolism processes. The expression levels of those proteins were changed under copper stimulation, but we are still not sure whether these candidates are eventually involved in the copper metabolism process, and if so, how they contributed to the WD phenotype was also largely unknown.

Although the differential expression of CTR2 was not detected in our iTRAQ analysis, the CTR2 p.S29L may also lead to the change of function of WT CTR2 even if no mutation-induced degradation happens. Although most of the CTR2 is localized in endosomes and lysosomes^23^, the biogenesis of the CTR1, the main Cu(I) influx transporter in mammalian cells, requires the existence of CTR2.^24^ CTR2 might modulate Cu(I) uptake by controlling the expression or function of CTR1, and primarily functions as a regulator of influx, but has a limited effect on Cu efflux.^35,36^ Here we reported a relative high patient frequency (6/191, 0.0314) of the CTR2 p.S29L (*SLC31A2* c.86C > T) variant in our cohort of 191 WD patients, and even higher in atypical WD patients (2/9, 0.222), suggesting a specific effect of CTR2 p.S29L on atypical WD patients without the classical *ATP7B* biallelic mutation. To date, no reports have indicated a direct association between CTR2 and WD, but our study indicated that CTR2 p.S29L may be an important pathogenetic variant in Chinese WD patients, especially in those with atypical disease-causing genotype of *ATP7B* gene. However, because of the limited number of the patients carrying CTR2 p.S29L variant, we were not able to observe if they had any differences in clinical manifestations with other WD patients. In our study, when incubated with CuSO_4_ solution, increased level of apoptosis was observed in CTR2^S29L^-overexpressed cell lines. These results suggest that the CTR2 p.S29L variant might cause more copper accumulation than does the WT protein, and that CTR2 p.S29L may contribute to the clinical manifestations of WD. However, whether CTR1 is also involved in this process remains unclear, and more in-depth molecular research is urgently needed.

## Data Availability

The data supporting the conclusions of this manuscript will be made available by the authors, without undue reservation, to any qualified researcher.
